# A State Nursing Service

**Published:** 1912-03-02

**Authors:** J. C. McWalter

**Affiliations:** Alderman, Dublin City. Dublin


### The Editor's Letter Box.

A STATE NURSING SERVICE.

*To the Editor of* THE HOSPITAL.

SIR,—The time may not yet be ripe for that free medical service at the cost of the State which was first advocated by my friend Professor Benjamin Moore, but I submit the time has already come for a free nursing service for the people. The cost would be trifling—half a million of money would provide 10,000 nurses at an average of £50 yearly each. This is nothing to a country which can spend twelve millions a year in old-age pensions and scarcely feel it. Ten thousand nurses would give about one to each 800 of the population—more than amply sufficient. I would include under the category midwives as well as medical and surgical nurses. Their services would be available on pretty much the same conditions as prevail for Jubilee nurses at present, except that they would not be confined to the poorer population. An immense impetus would be given to the nursing profession, because every capable young woman with a taste for it could be sure of a certain moderate competence. From this standing army of nursing sisters volunteers could always be found for special epidemic or military duties in any part of the Empire. Registration of all nurses would, of course, follow at once, and the pay being small, would not rob the voluntary hospitals of their best hands, but would be in the nature of a retaining fee sufficient to assure some degree of comfort for a nurse when nothing better was available. If the demand for a free nursing service is made loudly by the people they can get it.—Yours truly,

J. C. MCWALTER, M.A., M.D., LL.B.
Alderman, Dublin City.

Dublin, February 8.

